# New mindset in scientific method in the health field: Design Thinking

**DOI:** 10.6061/clinics/2015(12)01

**Published:** 2015-12

**Authors:** Fernando Kobuti Ferreira, Elaine Horibe Song, Heitor Gomes, Elvio Bueno Garcia, Lydia Masako Ferreira

**Affiliations:** Divisão de Cirurgia Plástica, Universidade Federal de São Paulo, São Paulo/SP, Brazil.

Society changes over time, as do companies, markets and forms of consumption. Gone are the days when having the lowest cost, best quality or highest market recognition would guarantee the survival of a company.

We live in a new world where innovation is a perceived value; and thus cannot be imposed by providers. Companies as well as people have the task of creating human being-centered relevant solutions. Solutions must be based on existing problems; thus, they must be desired and well received by users. Therefore, Design Thinking (DT) is becoming increasingly notorious.

DT is a new way of thinking and approaching issues; in other words, DT is a human being-centered model of thinking 1,2. The term *design* goes far beyond “aesthetics”, which is a term often related to *design*. *Design* does not simply mean the way things appear to be but also how they actually work. DT is based on three main pillars that support the herein discussed mindset, namely Empathy, Collaboration and Experimentation 3,4 (Figure 1).

*Empathy* involves our ability to understand the feelings or reactions from others by picturing ourselves in the same circumstances they are facing. Empathy requires diving into someone else’s world and knowing how they live, what they like and what their anxieties are, etc. The second pillar, *Collaboration*, involves working as a team with others to achieve a certain result or to participate in collective activities. Last but not least, *Experimentation* seeks to raise observations and experiences under different circumstances 3,4.

DT is widely applicable in the Health field to all actions directly or indirectly involving disease prevention and/or treatment. Therefore, adopting this method means applying an instrument that is essential to achieve real changes in society.

Several medical errors emerge from the little attention given to healthcare professionals, to patients’ real needs as well as to the way users interact with equipment, software, etc. For instance, traditional methods used during shift changes to exchange patient data from one health professional to another leads to situations that may cause miscommunication and misunderstandings. The various equipment in intensive care units competes for attention from health professionals by creating a cacophony that easily leads nurses and doctors to ignore alarms that signal real risk to patients’ lives 5.

Design is not just for designers. Professionals who launch health facilities may use DT to exponentially improve the experiences of health system users. DT has already been used to turn a cancer treatment center into a patient-centered facility. This transformation was based on the center creators’ *empathy* towards patients, their families and employees. Open interviews were used to obtain their *empathy* perspective and narrative responses were obtained from users and staff. Information was also collected from secondary sources such as articles found in the literature 6.

*Design* means understanding rather than assuming. Many experienced experts tend to assume that certain groups of people require certain solutions, although they do not understand the real needs of such groups. However, a good design is achieved by truly understanding users as well as the environment the users are in and by testing possible solutions. For instance, in 2010 Stanford University hired Jump Associates consulting firm to investigate why many of its doctors were exhausted and showed high stress levels at work (burnout syndrome). The consulting firm closely followed the doctors’ routines and performed in-depth interviews with many of them. They found an eight-month pregnant doctor who was accepting a greater than normal number of shifts. Although she was not required to work more than her colleagues did, she did not want to feel guilty for not working for a few weeks after her baby was born. In this case, the real problem was not the lack of maternity leave but rather the sense of guilt felt by doctors when they need to take leave for personal reasons. Thus, the consulting firm decided to focus on the organizational culture *design* and on campaigns to support doctors. If the team had only focused on improving the benefits provided to doctors, they would have taken initiatives in vain because these initiatives would not solve the real problem 5.

In contrast, Kaiser Permanent (KP), which is a leading US private hospital network, has a group of innovation-focused consultants who constantly use DT to improve network processes and systems. KP worked in partnership with the DT-based *design* company IDEO (Design and Innovation Consulting Firm) to improve nurses’ shift changes. Nurses often noted important clinical information on their own aprons or on loose papers. In addition, information exchange processes usually took 45 minutes or more, thus significantly delaying clinical activities. After applying the DT process, KP and IDEO developed the Nurse Knowledge Exchange. According to the Nurse Knowledge Exchange, medical information is exchanged using software with uncomplicated and standardized data entry at patient bedside; thus, allowing patients to participate in the process 7.

DT may be used in many other initiatives, such as in the prevention of acute infectious diseases. The Vienna Vaccine Safety Initiative - an international institution focused on promoting research and communication about vaccine safety – and the School of DT in Germany worked together using DT to answer the following question: “How can we lead doctors to encourage patients and their parents to prevent infectious diseases?” These institutions managed to implement a successful campaign 8.

The *Double Diamond* diagram was developed by the *Design Council* (UK) in 2005 as a simple way to graphically describe the DT process 9 (Figure 2).

The diagram is divided into four different phases, namely Discover, Define, Develop and Deliver, and aims to map the divergent and convergent stages of the design process by showing designers’ different ways of thinking.

The first phase in the Double Diamond model, “Discover”, marks the beginning of the project. This phase corresponds to a deep contextual dip into the challenge scenario. At this point, ethnographic techniques are used to understand how people live, work and relate to each other within the studied context:

- *Desk Research* (ethnography, market research, internal data analysis, e.g., the database containing data on the doctor’s patients, among others).
- *Shadowing* (in-person and/or virtual observation, e.g., groups on Facebook and WhatsApp*).*
- Interviews with users and *stakeholders.*
- Defining the service “Personas” (e.g., an insecure patient who calls the doctor by phone 10 times a day). In-depth interviews are held with these individuals to deeply understand them as well as to understand what they hear, say and think about the service.
- User's journey (a user’s mapped moments and activities).

The second phase in the Double Diamond model is called “Define,” which represents the definition phase, the moment when insights are refined. This phase aims to identify patterns and to reach conclusions based on collected data.

The main activities held during the “Define” phase are as follows: information affinity, essence problem definition (time, cost, etc.), information organization and intake (pause for observing the process as a whole).

The third phase in the Double Diamond model is known as “Develop,” which seeks to generate ideas and prototypes. The main activities and goals during the “Develop” phase comprise performing brainstorms with the team and end users (via SWAP, giving people 10 minutes to write their ideas on their own and then share them), defining the essence of the given ideas and comparing them to the core of the problems, defining the best idea(s) and creating prototypes.

The fourth and final phase in the Double Diamond model is called “Deliver,” which focuses on the adjustments and further refinements that must be performed to produce more mature prototypes in the medium and long term. The main activities and goals during this phase are testing, adjusting and validating the prototype.

The Double Diamond model is an abstract representation of what might happen within a project based on DT. However, the model should not be understood as a one-way flow. Thus, designers navigate the diamond phases; they intensify or abandon the use of tools and techniques and move back and forth as the challenge progresses.

Unlike the scientific method, which defines all the procedures before the project starts and gradually progresses into a one-way manner, designers using the Double Diamond model to innovate hardly follow a process with predictable inputs and outputs.

Figure 2 shows the intersection among the Double Diamond model phases and the DT pillars.

Traditional projects in academic study rely on the Cartesian scientific method, which holds an inside-out process. This method first launches a hypothesis and subsequently involves users in validation tests and procedures.

Differently, the DT is based on a humanistic approach. The outside-in process of DT is co-participatory and involves users from the very beginning. Combining this methodology and the traditional scientific methodology could improve the quality of studies in this field because the main focus is on the individual/patient/client/service. The DT methodology comes from meeting with advances in science and technology and the need to go beyond the frontiers aimed at developing products and services.

The final result is another major difference between the two methods. Researchers using traditional study methods seek to publish their papers in some high impact factor journal, whereas DT professionals seek solutions that aggregate and generate value and that can be quickly tested, validated and placed on the market or used for the patient’s benefit.

Therefore, the format of scientific work is diverse. Traditional scientific study requires following rules as well as the scientific quest guideline. In contrast, DT represents a straightforward process to consumers/patients. There is no need to write a 200-page dissertation in ABNT format, to summarize papers in the literature, or to insert appendices and attachments in order to ideate and implement an innovative solution. The difference lies in the practicality and in the immediate work with clients/patients.

User interviews, client/patient secret sharing, brainstorms, and post-it notes across the wall are some examples of DT activities. The final DT “product” is an idealized, prototyped, tested and validated solution reached by users/patients.

Currently, the world provides the same value to studies showing direct applicability to health; thus, these fundamental differences in thinking and conceptualization found in scientific studies require revision.

The Professional Master program aims at innovating by focusing on solving societal problems. This program will have a great methodological ally following the import of this tool into the strict sense Graduate level. DT is the most appropriate method to be used in scientific technological projects aimed at services that generate social, economic and political impact.

Properly integrating scientific technological projects requires an understanding of the cultural link between these two worlds (scientific and innovation), without incurring the error of creating tools featured as meaningless mixtures of existing templates in both knowledge fields. More thought about new perspectives emerged from the matching of these two approaches is necessary.

## Figures and Tables

**Figure 1:**
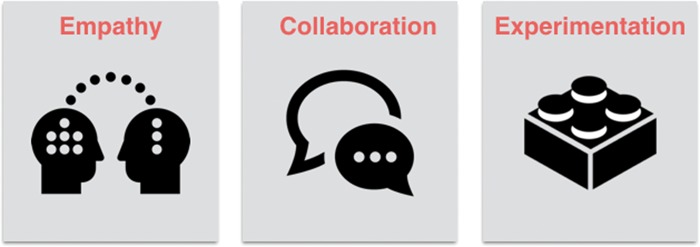
Design Thinking - main pillars.

**Figure 2:**
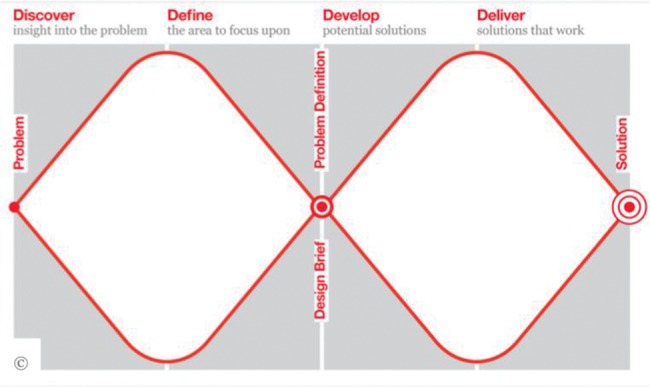
Double Diamond diagram – a graphical way of describing Design Thinking. http://www.designcouncil.org.uk/news-opinion/design-process-what-double-diamond
